# A nationwide study of breast reconstruction after mastectomy in patients with breast cancer receiving postmastectomy radiotherapy: comparison of complications according to radiotherapy fractionation and reconstruction procedures

**DOI:** 10.1038/s41416-024-02741-4

**Published:** 2024-06-05

**Authors:** Hyejo Ryu, Kyung Hwan Shin, Ji Hyun Chang, Bum-Sup Jang

**Affiliations:** 1 Department of Radiation Oncology, Chung-Ang University Gwangmyeong Hospital, Gwangmyeong, Korea; 2 Department of Radiation Oncology, College of Medicine, Seoul National University, Seoul, Korea; 3 Department of Radiation Oncology, Seoul National University Hospital, Seoul, Korea; 4 Institute of Radiation Medicine, Seoul National University Medical Research Center, Seoul, South Korea

**Keywords:** Breast cancer, Radiotherapy, Reconstruction

## Abstract

**Background:**

We examined the patterns of breast reconstruction postmastectomy in breast cancer patients undergoing postmastectomy radiotherapy (PMRT) and compared complications based on radiotherapy fractionation and reconstruction procedures.

**Methods:**

Using National Health Insurance Service (NHIS) data (2015–2020), we analysed 4669 breast cancer patients with PMRT and reconstruction. Using propensity matching, cohorts for hypofractionated fractionation (HF) and conventional fractionation (CF) were created, adjusting for relevant factors and identifying grade ≥3 complications.

**Result:**

Of 4,669 patients, 30.6% underwent HF and 69.4% CF. The use of HF has increased from 19.4% in 2015 to 41.0% in 2020. Immediate autologous (32.9%) and delayed two-stage implant reconstruction (33.9%) were common. Complication rates for immediate (*N* = 1286) and delayed two-stage (*N* = 784) reconstruction were similar between HF and CF groups (5.1% vs. 5.4%, *P* = 0.803, and 10.5% vs. 10.7%, *P* = 0.856, respectively) with median follow-ups of 2.5 and 2.6 years. HF showed no increased risk of complications across reconstruction methods.

**Conclusion:**

A nationwide cohort study revealed no significant difference in complication rates between the HF and CF groups, indicating HF for reconstructed breasts is comparable to CF. However, consultation regarding the fractionation for reconstructed breast cancer patients may still be necessary.

## Introduction

The escalating incidence of breast cancer has led to a corresponding rise in the number of breast cancer patients opting for breast reconstruction. This trend is particularly evident in South Korea, where breast reconstruction surgery following mastectomy gained coverage under the National Health Insurance Service (NHIS) in April 2015. Consequently, the rate of breast reconstruction cases in South Korea rose from 19.4% in 2015 to 53.4% in 2018 [1]. Similarly, the number of patients requiring postmastectomy radiotherapy (PMRT) is also expected to increase, given that PMRT is administered to one-third of breast cancer patients [2]. Despite the growing number of patients undergoing breast reconstruction and PMRT, the question of determining the optimal approach for patients receiving PMRT remains unsolved.

PMRT is associated with reconstruction-related complications and negatively impacts patients’ satisfaction. According to the Mastectomy Reconstruction Outcomes Consortium (MROC), PMRT significantly elevated the risk of complications by 2.6 times higher in patients with implant-based reconstructions compared to those who did not receive PMRT [3]. Likewise, irradiated autologous flaps exhibited a stronger association with fat necrosis and revision surgery compared to unirradiated flaps [4]. Patients with implant reconstruction and PMRT reported the lowest satisfaction levels [3]. These findings highlight the importance of finding strategies to minimise reconstruction-related complications.

Breast reconstruction is a multifaceted procedure that encompasses a variety of options, making it difficult to reach a consensus on the integration of reconstruction and PMRT. There are several approaches for breast reconstruction in terms of timing and materials. The timing of reconstruction can be classified as immediate, delayed, or a combination of both (delayed two-stage) [5]. In delayed two-stage reconstruction, known as delayed-immediate, a tissue expander is placed immediately with mastectomy, and it is exchanged for the implant or autologous tissues later [6]. Reconstruction materials can be either implants or autologous tissue such as fat or muscle, each with its unique advantages and drawback. While autologous flaps exhibit greater tolerance to irradiation compared to implants, not all patients are eligible for autologous reconstruction.

The advancements in radiation techniques have also added to the complexity of PMRT in reconstructed breasts. One of the major changes is the adoption of hypofractionation in breast cancer radiotherapy. By delivering a higher dose per fraction, HF can reduce treatment duration. Apart from convenience, HF has demonstrated comparable oncological outcomes to conventional fractions while exhibiting similar or even superior toxicity results [7–10]. However, these studies excluded patients with breast reconstruction. Consequently, the use of hypofractionation for reconstructed breasts remains controversial. For instance, the ESTRO guideline recommends adopting hypofractionation regardless of any oncoplastic surgery, while the ASTRO guideline does not yet endorse it [11, 12].

Therefore, we conducted a nationwide study to examine the current practices in breast reconstruction with PMRT following the implementation of insurance reimbursement in 2015. In addition, we compared the complication rates associated with hypofractionation and conventional fractionated PMRT. Ultimately, this study aimed to provide comprehensive nationwide data on breast reconstruction approaches and evidence for the comparable safety of HF for patients undergoing breast reconstruction.

## Materials and methods

### Data sources and study population

In South Korea, medical services in South Korea are covered by the NHIS, so it harbours large datasets of healthcare utilisation on a nationwide scale. It can provide demographic information, death information, and medical services utilisations at outpatient clinics as well as admissions. For research purposes, any researcher with ethical approval from the institutional review board can request the database. The personal information is deidentified, and analysis can be performed by using the International Statistical Classification of Diseases and 10th revision (ICD-10), and a procedure or operation code.

The study was approved by the Seoul National University Hospital’s Institutional Review Board (Number 2204-067-1315). The need for informed consent was waived due to the use of pseudonymized data. We requested a customised NHISS database of Korean female breast cancer patients who underwent mastectomy and postmastectomy radiation therapy (PMRT) between 2015 and 2020. The operational codes used for this study are listed in Supplementary Table 1. The study period was between 2015 and 2021. This period was chosen to capture the impact of the reimbursement for breast reconstruction, which began in 2015. Among the 58,028 women with mastectomy and radiation therapy for breast cancer (ICD-10; C50) between 2015 and 2020, we initially excluded no history of reconstruction (*n* = 6020) (Supplementary Fig. S1). Next, we excluded patients with multiple mastectomies (*n* = 925), time interval longer than 1 year between mastectomy and PMRT (*n* = 7950), and PMRT number less than 14 or greater than 35 (*n* = 38,464).

Finally, there were 4669 patients eligible for a pattern of care analysis.

### Definitions

PMRT was administered following mastectomy surgery. Information on fraction size or number was unavailable in the database; therefore, the number of claim codes for radiation treatment, including chest wall and tumour bed boost irradiation, was considered to represent the fraction number. Only claim codes recorded up to three months from the initial PMRT date were counted. Using the survey result of the pattern of care study conducted by the Korean Radiation Oncology Group, we divided fractionation groups as follows [2]. Conventional fractionation (CF) was defined as 25–35 radiation treatments, assuming a total dose of at least 45-50 Gy in 1.8–2.0 Gy fractions. Conversely, hypofractionation (HF) was defined as 14 to 24 radiation treatments, assuming a total dose of 40–42.5 Gy in fractions greater than 2 Gy.

Breast reconstruction can be classified based on the reconstruction material and timing. Autologous breast reconstruction utilises tissue from the patient’s own body, such as latissimus dorsi (LD), transverse rectus abdominis musculocutaneous (TRAM), or deep inferior epigastric artery perforator (DIEP). Implant-based involves the placement of breast implants either a tissue expander (TE) or direct-to-implant (DTI). Regarding the timing of reconstruction, immediate reconstruction is performed on the same day as mastectomy or before the initiation of PMRT. Delayed reconstruction consisted of two types of reconstruction: one-stage and two-stage delayed reconstruction. One-stage delayed reconstruction involves reconstruction surgery after the completion of PMRT, while two-stage delayed reconstruction involves tissue expander insertion followed by PMRT and final definitive reconstruction.

The primary outcome of this study was the incidence of reconstruction-related complications requiring surgery following PMRT. Only surgeries performed by the Department of Plastic Surgery were considered complication events. We employed the operational codes for breast capsulectomy (breast capsulorrhaphy, capsulotomy, capsular flap), debridement, and skin graft. Capsulectomy performed on the day of final reconstruction was not considered a complication. Capsular contracture referred to capsulectomy performed exclusively in patients with implants. The capsular contracture was limited to patients requiring surgical intervention, because the specific procedure code for breast capsulectomy assigned by the national standard code was available. Wound complication was defined as an adverse event necessitating surgical debridement and skin graft. Patients with delayed two-stage complications were excluded if they lacked a final reconstruction history or complication history.

### Statistical analysis

Basic descriptive statistics were used to examine the practice pattern trend in PMRT and breast reconstruction. The cohort was divided based on the timing of breast reconstruction (immediate vs. two-stage delayed reconstruction), and the toxicity outcomes between CF and HF were compared. Baseline characteristics between CF and HF were evaluated using t-tests and chi-square tests for continuous and categorical variables, respectively. To adjust the baseline characteristics between two fractionated regimens, a 1:1 greedy nearest propensity matching method was implemented in PSMATCH (SAS version 9.4) with a caliper of 0.2. Adjusted covariates included age, hypertension, diabetes, reconstruction material, IMRT, year of PMRT, and bolus. A standardised mean difference (SMD) of 0.2 was set as the cut-off value between the groups.

Crude complication rates between the two fractionation groups were compared using chi-squared tests. To account for the difference in the follow-up duration, a competing risk analysis for complications was performed, with death as the competing risk. Cox regression model was employed to identify variables associated with complications. Variables with a *P* value < 0.1 in univariate analysis were included in the multivariate analysis. The follow-up duration was defined as the time interval between the first date of PMRT and the date of the complication event, death, or the last visit. Categorical variables were represented as proportions with percentages. Continuous variables were expressed as medians with range. Data processing and statistical analyses were performed by Statistical Analysis System (SAS) version 9.4 (SAS Institute, Cary, NC, USA) and Stata version 15.0 (StataCorp, College Station, TX).

## Results

### Practice patterns of postmastectomy radiotherapy and breast reconstruction

**Fig. 1:**
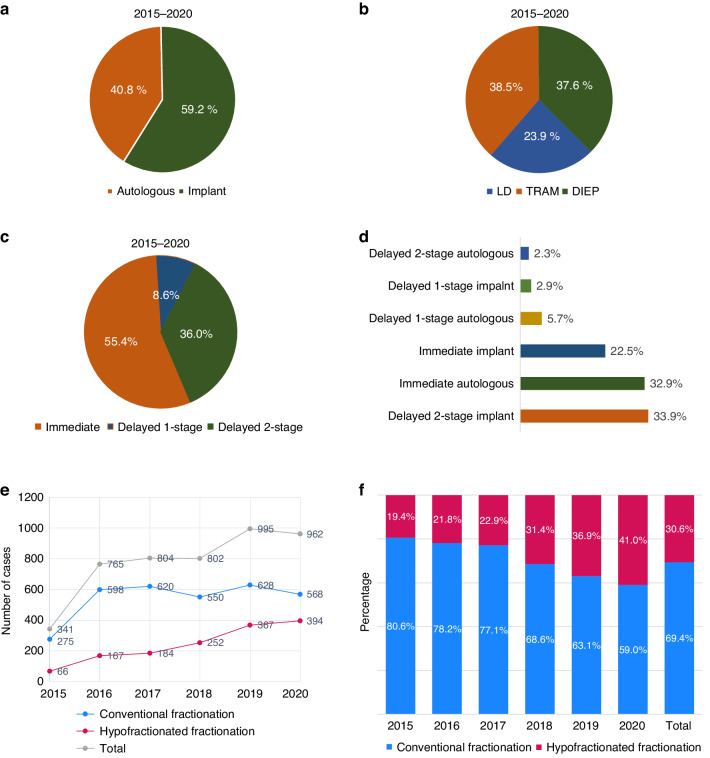
Types of breast reconstruction and fractionations in patients with postmastectomy radiotherapy (PMRT) between 2015 and 2020. **a** Rate of implant and autologous-based reconstructions. **b** Materials used for autologous reconstruction. **c** Types of breast reconstructions by timing. **d** Breast reconstruction by timing and material. **e** Rate of conventional fractionation and hypofractionated fractionation. **f** The yearly trend of fractionation for reconstructed breasts between 2015 and 2020.*LD* lattissimus dorsi, *TRAM*transverse rectus abdominis musculocutaneous, *DIEP* deep inferior epigastric artery perforator

A significant increase in the utilisation of breast reconstruction and PMRT was observed, with the number of patients undergoing these procedures rising from 341 in 2015 to 962 in 2020. Among 4,669 breast cancer patients who received PMRT and reconstruction between 2015 and 2020, implants were employed in 59.2% of the cases (Fig. 1a). Within the realm of autologous reconstruction, TRAM flaps were the most prevalent choice (38.5%), followed by DIEP (37.6%) and LD (23.9%) (Fig. 1b). Immediate reconstruction was the most frequently performed timing strategy, accounting for 55.4% of patients. Delayed two-stage reconstruction was utilised in 36.0% of patients, followed by delayed one-stage reconstruction in 8.6% of patients (Fig. 1c). When considering both reconstruction materials and timing, delayed two-stage implant reconstruction was the most common procedure, performed in 33.9% of the cohort. Immediate autologous reconstruction and immediate implant reconstruction followed with respective frequencies of 32.9% and 22.5% (Fig. 1d). Regarding fractionation, 69.4% of the cohort received conventional fractionation (CF), while 30.6% underwent hypofractionation (HF). Notably, a gradual increase in the use of HF was observed, with the proportion of patients receiving HF rising from 19.4% (66 cases) in 2015 to 41.0% (394 cases) in 2020 (Fig. 1e, f).

### Immediate breast reconstruction: baseline characteristics and complication incidence rate

A total of 2436 patients with immediate reconstruction were eligible for the complication analysis. Prior to propensity matching, there were 1793 patients in the CF group and 643 patients in the HF group. Table 1 summarises the baseline characteristics of the immediate reconstruction cohort before and after matching. In the unmatched cohort, HF was associated with recent year of PMRT, IMRT technique, and shorter follow-up periods. The proportion of implant use was marginally higher in the CF group. Significantly more patients in the CF group received bolus treatment. Following 1:1 matching, all variables except PMRT fraction number were well-balanced. The median PMRT fraction number of CF and HF patients was 28 and 17, respectively.

**Table 1:** Baseline characteristics of the immediate reconstruction cohort before and after propensity matching.

	Before 1:1 matching				After 1:1 matching		
	CF	HF	SMD	CF		HF	SMD
Characteristics	*N* = 1793	*N* = 643		*N* = 643		*N* = 643	
Year							
2018–2020	998 (55.7)	484 (75.3)	0.397	473 (73.6)		484 (75.3)	0.050
2015–2017	795 (44.4)	159 (24.7)		170 (26.4)		159 (24.7)	
Age							
>45	963 (53.7)	352 (54.7)	0.022	351 (54.6)		352 (54.7)	0.009
≤45	830 (46.3)	291 (45.3)		292 (25.4)		291 (45.3)	
Hypertension							
Yes	450 (25.1)	153 (23.8)	−0.063	159 (24.7)		153 (23.8)	−0.058
No	1343 (74.9)	490 (76.2)		484 (75.3)		490 (76.2)	
Diabetes							
Yes	549 (30.6)	186 (28.9)	-0.071	187 (29.1)		186 (28.9)	−0.022
No	1244 (69.4)	457 (71.7)		643 (70.9)		457 (71.7)	
Fraction number	28 (25–35)	17 (15–24)	−3.612	28 (25–35)		17 (15–24)	−3.517
IMRT							
Yes	471 (26.2)	453 (70.5)	0.972	453 (70.5)		453 (70.5)	0
No	1322 (73.7)	190 (29.5)		190 (29.5)		190 (29.5)	
Bolus							
Yes	540 (30.1)	147 (22.9)	-0.190	146 (22.7)		147 (22.9)	0.003
No	1253 (69.9)	496 (77.1)		497 (77.3)		496 (77.1)	
Reconstruction material							
Implant	751 (41.9)	243 (37.8)	−0.025	268 (41.7)		243 (37.8)	−0.021
Autologous	1042 (58.1)	400 (62.2)		375 (58.3)		400 (62.2)	
Median follow-up (year)	3.3 (0.3–6.6)	2.5 (0.3–5.9)	−0.357	2.5 (0.3–5.9)		2.5 (0.3–5.9)	−0.041

*CF* conventional fractionation, *HF* hypofractionated fractionation, *SMD* standardised mean difference, *IMRT* intensity modulating radiotherapy, *mo* months.

**Fig. 2:**
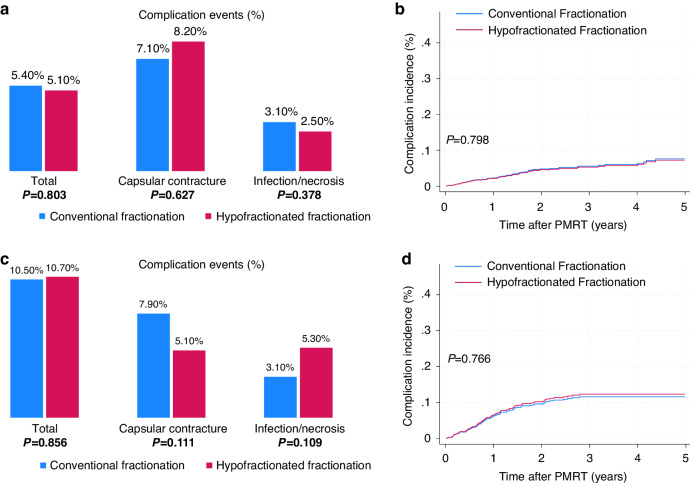
Complications events and incidence according to fractionation and reconstruction timing. **a** Complication events in the matched immediate reconstruction cohort (*N* = 1286). **b** Cumulative incidence in the matched immediate reconstruction cohort (*N* = 1286). **c** Complication events in the matched two-stage delayed reconstruction cohort (*N* = 784). **d** Cumulative incidence in the matched two-stage delayed reconstruction cohort (*N* = 784).

The matched cohort analysis revealed comparable follow-up durations between the CF and HF groups. Both groups had a median follow-up of 2.5 years, with a range of 0.3–5.9 years. During a median follow-up duration of 2.5 years, the complication rates were 5.4% in CF and 5.1% in HF (*P* = 0.803). The complication incidence was not significantly different between two groups (Fig. 2a). Capsular contracture was the most common severe complication, occurring 7.1% in CF and 8.2% in HF (*P* = 0.627). The wound complications requiring debridement and skin graft were observed in 3.1% in CF and 2.5% in HF (*P* = 0.378). Figure 2b illustrates the cumulative incidence of complications, and the curves of the two fractionation groups did not differ from each other (*P* = 0.798). Similarly, there was no difference in complication incidence in the unmatched cohort as well (Supplementary Fig. S2A). In the matched cohort, implant-based immediate reconstruction was the only prognostic factor significantly associated with complications (hazard ratio (HR) 2.65, confidence interval (CI) 1.72–4.07, *P* < 0.001, Table 2). However, HF, IMRT, and bolus were not identified as significant factors.

**Table 2:** Cox regression proportional hazards model for complication in the immediate reconstruction cohort after matching (*N* = 1286).

	Univariate			Multivariate	
Characteristics		Hazard ratio (95% CI)	*P*	Hazard ratio (95% CI)	*P*
Year	2018–2020 (vs. 2015–2017)	1.06 (0.66–1.71)	0.784		
Age	>45 (vs. ≤45)	1.03 (0.63–1.65)	0.913		
Hypertension	Yes (vs. no)	1.89 (0.97–1.75)	0.062	1.75 (0.87–3.45)	0.116
Diabetes	Yes (vs. no)	1.06 (0.63–1.78)	0.833		
Fractionation	HF (vs. CF)	1.01 (0.67–1.53)	0.832		
IMRT	Yes (vs. no)	1.16 (0.74–1.80)	0.520		
Bolus	Yes (vs. no)	1.24 (0.78–1.95)	0.361		
Reconstruction material	Implant (vs. autologous)	2.70 (1.76–4.14)	<0.001	2.65 (1.72–4.07)	<0.001

*HF* hypofractionated fractionation, *CF* conventional fractionation, *IMRT* intensity modulating radiotherapy, *mo* months.

### Delayed two-stage breast reconstruction: baseline characteristics and complication incidence rate

In a retrospective analysis of 1347 patients undergoing delayed two-stage breast reconstruction, comprising 782 in the CF group and 565 in the HF group, the baseline characteristics of the unmatched and matched cohorts were compared (Table 3). Like the immediate breast reconstruction cohort, discrepancies were observed in the year of treatment, fraction number, intensity-modulated radiotherapy (IMRT) utilisation, bolus administration, and follow-up duration. The differences in the baseline characteristics were similar to the immediate cohort. Following propensity matching, the variables, except PMRT fraction number, were balanced between in the two groups. The median PMRT fraction number of CF and HF was 28 and 17, respectively. Unlike the immediate reconstruction cohort, implants were predominately used as the final reconstruction material. The median interval between PMRT and final reconstruction was 258 days (range 31–985 days).

**Table 3:** Baseline characteristics of the delayed two-stage reconstruction cohort before and after propensity matching.

	Before 1:1 matching			After 1:1 matching		
	CF	HF	SMD	CF	HF	SMD
Characteristics	*N* = 782	*N* = 565		*N* = 392	*N* = 392	
Year						
2018–2020	500 (63.9)	415 (73.5)	0.399	288 (73.5)	295 (75.3)	0.109
2015–2017	282 (36.1)	150 (26.5)		104 (26.5)	97 (24.7)	
Age						
>45	387 (49.5)	278 (49.2)	−0.022	188 (48.0)	200 (51.0)	0.090
≤45	395 (50.5)	287(50.8)		204 (52.0)	192 (49.0)	
Hypertension						
Yes	173 (22.1)	121 (21.4)	0.063	84 (21.4)	82 (20.9)	-0.058
No	609 (77.9)	444 (78.6)		308 (78.6)	310 (79.1)	
Diabetes						
Yes	176 (22.5)	145 (25.7)	0.071	87 (22.2)	103 (26.3)	0.109
No	606 (77.5)	420 (74.3)		305 (77.8)	289 (73.7)	
Fraction number	28 (25–35)	17 (15–24)	-4.504	28 (25–35)	17 (15–24)	−4.426
IMRT						
Yes	260 (33.2)	428 (75.8)	1.009	251 (64.0)	256 (65.3)	0.043
No	522 (66.8)	137 (24.2)		141 (36.0)	136 (34.7)	
Bolus						
Yes	262 (33.5)	127 (22.5)	-0.337	114 (29.1)	101 (25.8)	-0.199
No	520 (66.4)	438 (77.5)		278 (70.9)	291 (74.2)	
Reconstruction material						
Implant	723 (92.5)	532 (94.2)	0.165	369 (94.1)	372 (94.9)	0.091
Autologous	59 (7.5)	33 (5.8)		23 (5.9)	20 (5.1)	
Median follow-up (year)	3.2 (0.9–6.4)	2.6 (0.4–6.3)	−0.269	2.8 (0.9–6.1)	2.6 (0.6–6.0)	−0.126

*CF* conventional fractionation, *HF* hypofractionated fractionation, *SMD* standardised mean difference, *IMRT* intensity modulating radiotherapy.

The matched cohort had a median follow-up duration of 2.6 years (0.6–6.1 years), with the CF group at 2.8 years (0.9–6.1 years) and the HF group at 2.6 years (0.6–6.0 years). The overall incidence of complication did not differ significantly between the groups (CF: 10.5% and HF: 10.7%, *P* = 0.856) (Fig. 2c). Capsular contracture occurred in 7.9% and 5.1% in CF and HF, respectively (*P* = 0.111). Similarly, the wound complication rates did not differ significantly between the two groups (CF: 3.1% and HF: 5.3%, *P* = 0.109). Furthermore, the cumulative complication incidence did not differ between the two groups (*P* = 0.766) (Fig. 2d). This was also consistent in the unmatched group was observed as well (Supplementary Fig. S2B). Univariate and multivariate analyses revealed hypertension (HR 2.42, CI 1.39–3.5, *P* = 0.002) and diabetes (HR 2.36, CI 1.36–4.01, *P* = 0.002) to be significantly associated with an increased risk of complications (Table 4). Except for patients’ comorbidities, the factors related to radiation or reconstruction did not increase the risk of complications. Neither the use of bolus nor the time interval between PMRT and definitive surgery demonstrated prognostic significance.

**Table 4:** Cox regression proportional hazards model for complication in the delayed two-stage reconstruction cohort after matching (*N* = 784).

	Univariate			Multivariate	
Characteristics		Hazard ratio (95% CI)	*P*	Hazard ratio (95% CI)	*P*
Year	2018–2020 (vs. 2015–2017)	1.08 (0.68–1.69)	0.736		
Age	>45 (vs. ≤45)	1.02 (0.95–1.01)	0.190		
Hypertension	Yes (vs. no)	1.75 (1.10–2.76)	0.016	2.42 (1.39–3.50)	0.002
Diabetes	Yes (vs. no)	2.10 (1.37–3.23)	0.001	2.36 (1.36–4.01)	0.002
Fractionation	HF (vs. CF)	1.05 (0.69–1.61)	0.793		
IMRT	Yes (vs. no)	0.75 (0.49–1.15)	0.196		
Bolus	Yes (vs. no)	1.25 (0.82–1.91)	0.290		
Reconstruction material	Implant (vs. autologous)	1.14 (0.45–2.88)	0.779		
Interval between PMRT and definitive surgery	10 mo (vs. ≤10 mo)	0.99 (0.99–1.00)	0.141		

*HF* hypofractionated fractionation, *CF* conventional fractionation, *IMRT* intensity modulating radiotherapy, *mo* months.

## Discussion

This study represents the first population-based national cohort study to investigate the pattern of care and complications among patients with breast reconstruction and PMRT between 2015 and 2020. Despite the increasing number of breast reconstructions, uncertainties persist regarding the timing and materials employed in the context of PMRT. Moreover, with advancements in radiation technology, hypofractionation has emerged as a replacement for conventional fractionation in breast cancer treatment. However, despite several randomised prospective studies, the impact and safety of hypofractionation under breast reconstruction setting was not well studied. Here, we utilised the big data from the National Health Insurance Service and reported the current status of breast reconstruction and PMRT in the South Korean population and showed comparable complication rates regardless of fractional regimens.

Our nationwide cohort study revealed that immediate reconstruction (55.4%) was the most preferred approach, followed by delayed two-stage reconstruction (36.0%). Within immediate reconstruction, autologous tissues were preferentially selected over implants. This finding aligns with the concerns regarding the elevated complications risk associated with implants compared to autologous tissue in an immediate reconstruction setting. Despite these concerns, implants remained a population choice, probably due to the shorter surgery time, faster recovery, and no donor site morbidity. Delayed two-stage reconstruction with implants was also commonly performed in our cohort. This approach is endorsed by the NCCN, as the immediate implant placement carries an increased incidence of capsular contracture, malposition, and poor cosemesis [13]. However, delayed reconstruction using autologous tissue was rarely chosen compared to the immediate reconstruction setting.

We found comparable toxicity between CF and HF in the immediate reconstruction cohort [14]. Globally, a limited number of studies have investigated the use of HF in reconstructed breasts, with heterogeneity in fractionation regimen and complication rates. Another cohort study conducted in Brazil examined the safety of hypofractionated PMRT (2.65–2.67 Gy/fraction) in reconstructed breasts. Among 35 patients who underwent implant-based immediate reconstruction, capsular contracture developed in 11.4% [14]. In contrast, a U.S. Phase II trial reported an incidence of severe complications of 35% in 43 patients receiving a dose of 36.63 Gy in 11 fractions with a daily fraction size of 3.33 Gy [15].

Beyond compatibility between fractionation regimens, our study identified several associated risk factors for reconstruction-related complications. Implant usage emerged as the sole significant prognostic factor for complications. This aligns with existing knowledge regarding the increased susceptibility of implants to radiation-induced complications. Despite the elevated risk of adverse effects such as capsular contracture associated with implants, their popularity persists due to cost-effectiveness and broader eligibility compared to autologous tissues. Consequently, further advancements in implant-based reconstruction and radiation techniques are paramount to enhancing reconstruction outcomes. While bolus application has been shown to increase toxicity in reconstructed breasts, our study did not identify a significant association between bolus usage and complication risk. This discrepancy may arise from the application of different bolus definitions in our study and the prior literature. Bolus is more likely to induce skin-related toxicity, whereas our study focused primarily on complications requiring surgical interventions [16]. However, de Sousa et al. reported an increased risk of infection (HR 10.3, CI 1.7–61.8), and reconstruction failure (HR 13.89, CI 2.24–85.98) in patients undergoing two-stage reconstructions when bolus was applied daily [17]. In contrast, alternate-day bolus usage was not associated with complication risk. Therefore, it is still important that the decision to use bolus for PMRT should be individualised considering the benefits and the risks.

A recent prospective randomised trial, the Radiation Fractionation on Patient Outcomes After Breast REConstruction (FABREC) study, found no significant differences in oncologic outcomes or chest wall toxicity between conventional fractionation and hypofractionation in 400 patients with immediate implant-based reconstructions [18]. The FABREC trial also reported improved quality of life in patients over 45 years old, along with reduced treatment breaks and financial toxicity in the HF group. Another pivotal study, RT CHARM (ALLIANCE A221505), is currently underway and includes both immediate and delayed reconstruction. Its results are eagerly anticipated, as they will provide valuable data on the safety of hypofractionation in the reconstruction setting.

Addressing the role of acellular dermal matrix (ADM) in immediate implant-based breast reconstruction is vital due to its widespread use and potential impact on complications. ADM is a biomaterial processed to remove cellular components while preserving extracellular matrix structure. Until now, the effect of ADM on patients undergoing PMRT is not well defined, but a few retrospective studies suggested a protective role in irradiated patients [19–21]. However, our study could not directly identify its actual usage because it has not been reimbursed by the government. A multi-institutional data from the South Korea Radiation Oncology Group indicated its prevalence, with over 50% utilisation in breast reconstruction and PMRT between 2015 and 2016 [22]. Considering the contemporary reliance on ADM in breast reconstruction practices, it may be reasonable to assume its significant incorporation in our study population, especially since ADM is routinely integrated into implant-based reconstruction protocols at our institution.

In addition to ADM usage, it is also important to understand the dosimetric planning and target delineation in our population. While the specific planning protocols may vary among hospitals in South Korea, it seems to follow the general principle [23]. For the hypofractionation scheme, at least 95% planning target volume was required to be covered by 95% of the prescribed dose, and the maximum point dose (D max) was limited to below 105–107%. Organ-at-risk constraints ensured that the volume of the ipsilateral lung receiving 5 Gy (V5) and 20 Gy (V20) did not surpass 45% and 20%, respectively. The mean dose to the heart was maintained be low 3 Gy and 5 Gy for right-sided tumours and left-sided tumours, respectively. The most commonly utilised RT techniques were forward IMRT (field-in-field) followed by VMAT and 3D conformal, according to a multi-institutional retrospective study conducted in South Korea [22]. In terms of target delineation, it seems that there is no specific preference between RTOG and ESTRO guidelines among radiation oncologists in South Korea [23]. Also, some institutions adopted new ESTRO-ACROP target delineation guideline which allows excluding implants from the clinical target volume [24, 25]. As there is no information on dosimetry planning and target delineations, it will be critical to interpret our results with caution.

Our study has several limitations. In our study, we excluded patients who received fewer than 14 or more than 35 fractions of radiotherapy, which could have arisen from various causes such as multiple treatment courses, treatment for metastatic lesions, or unconventional fractionation regimens. Furthermore, there may have been discrepancies between the actual number of fractions delivered and the number billed due to variations in billing practices and treatment principles across institutions. While it is challenging to derive precise radiation fractionation details from such a large-scale dataset, we believe existence of a significant difference in radiation fractions between the HF and CF groups presented, which is supported by several factors: First, the trend of increasing HF adoption in Korea since 2015 aligns with the observed pattern in our study [26]. Second, a separate analysis by Kim et al. using individual patient (*N* = 393) revealed comparable major breast complication rates (12.0% vs. 12.3%) following immediate reconstruction for both HF and CF groups [27]. While we recognise the inherent limitations of the nationwide database [28], we believe our study serves as a valuable step towards establishing a more robust and verifiable method for radiation fractionation assessment within national databases. Accurately filtering out such complexities from large datasets proved exceedingly challenging, necessitating an exclusion approach to maintain cohort homogeneity. Although this resulted in a reduced sample size, the enhanced treatment homogeneity within the cohort enabled us to draw more meaningful conclusions from our analysis. The radiation field or site could not be identified in the national database. Our definition of complications was limited to major events requiring capsulectomy, wound debridement, or skin graft by plastic surgeons. Other complications requiring only medical treatment were not included. Furthermore, the relatively short follow-up duration in some patients may have resulted in the failure to capture adverse events. Therefore, it is necessary to approach the interpretation of our findings with caution.

## Conclusion

This nationwide study, the first to examine trends and complications in breast cancer patients undergoing both reconstruction and PMRT, revealed a moderate increase in the adoption of HF. Importantly, the major complication rates remained comparable between HF and CF, irrespective of the timing of reconstruction. Nevertheless, ongoing prospective trials on fractionation hold promise for further optimising PMRT strategies in this setting.

### Supplementary information

Supplementray information

## Data Availability

Any researcher with an approved IRB can request a customised database to the National Health Insurance Data Sharing Service (https://nhiss.nhis.or.kr/). Upon approval, all raw data were only accessible from “Data analysis room” located within the National Health Insurance Service (NHIS-2022-1-675).
