# Supplementary material for: A nationwide study of breast reconstruction after mastectomy in patients with breast cancer receiving postmastectomy radiotherapy: comparison of complications according to radiotherapy fractionation and reconstruction procedures
Source: Br J Cancer. 2024 Jun 5;131(2):290–8. doi: 10.1038/s41416-024-02741-4 (PMC11263609; doi:10.1038/s41416-024-02741-4)

# **Supplementary Information**

## Supplementary Table S1. A list of study codes

| Procedure codes | Codes |
| --- | --- |
| Total mastectomy (with ALND) | N7138 |
| Total mastectomy (without ALND) | N7139 |
| Using autologous tissue |  |
| Autologous-LD flap | N7140 |
| Autologous-muscle-sparing LD myocutaneous flap | N7141 |
| Autologous-extended LD myocutaneous flap | N7142 |
| Autologous-pedicled TRAM flap | N7143 |
| Autologous-bipedicled TRAM flap | N7144 |
| Autologous-transverse TRAM free flap | N7145 |
| Autologous-muscle sparing TRAM free flap | N7146 |
| Autologous-DIEP | N7147 |
| Using Implant |  |
| Breast tissue expander insertion (2 stage breast reconstruction, first stage) | N7148 |
| Implant -Immediate insertion of permanent breast Implant | N7149 |
| Implant -Insertion of permanent implant following breast tissue expansion (2 stage breast reconstruction, first stage) | N7150 |
| Teletherapy | HD051, HD052, HD053, HD054, HD055,HD056 |
| Rotational Irradiation | HD057,HD058,HD059 |
| 3-Dimensional Radiation therapy | HD061 |
| Intensity modulating Radiation therapy | HZ271 |
| Bolus | HD032 |
| Suture of the wound including debridement | SC021-027 |
| Breast Capsulectomy (Breast Capsulorrhaphy, Capsulotomy, Capsular Flap) | N7151 |
| Infectious wound dressing | M0121 |
| Skin graft | S0175 |
| Disease codes (ICD-10) | **Codes** |
| Breast cancer | C50 |
| Hypertension | I10, I11, I12, I13, I15 |
| Diabetes mellitus | E10, E11, E12, E13, E14 |

Abbreviation: ALND, Axillary lymph node dissection; LD, Latissimus dorsi; TRAM, Transverse rectus abdominis myocutaneous; DIEP, deep inferior epigastric artery perforator; ICD-10, International Classiﬁcation of Diseases, 10th Revision

## Supplementary Table S2. Cox regression proportional hazards model for complication in the immediate reconstruction cohort before matching (N=2,436)

|  | Univariate | |  | |  | Multivariate | | | |  | |  |
| --- | --- | --- | --- | --- | --- | --- | --- | --- | --- | --- | --- | --- |
| Characteristics |  | **Hazard Ratio**  **(95% CI)** | | **P** | | | **Hazard Ratio**  **(95% CI)** | **P** | | |  |  |
| Year | 2018-2020  (vs. 2015-2017) | 1.23 (0.85-1.78) | | 0.270 | | |  |  | | |  |  |
| Age | > 45  (vs. ≤ 45) | 0.98 (0.69-1.38) | | 0.901 | | |  |  | | |  |  |
| Hypertension | Yes  (vs. No) | 1.34 (0.91-1.95) | | 0.136 | | | 1.41 (0.96-2.05) | 0.120 | | |  |  |
| Diabetes | Yes  (vs. No) | 0.95 (0.65-1.39) | | 0.795 | | |  |  | | |  |  |
| Fractionation | HF  (vs. CF) | 1.07 (0.72-1.61) | | 0.718 | | |  |  | | |  |  |
| IMRT | Yes  (vs. No) | 1.23 (0.63-1.37) | | 0.267 | | |  |  | | |  |  |
| Bolus | Yes  (vs. No) | 0.93 (0.03-1.37) | | 0.706 | | |  |  | | |  |  |
| Reconstruction material | Implant  (vs. Autologous) | 3.41 (2.38-4.76) | | <0.001 | | | 3.57 (2.38-5.26) | | <0.001 | | | |

Abbreviation: HF, Hypofractionated fractionation; CF, Conventional fractionation; IMRT, Intensity modulating radiotherapy

## Supplementary Table S3. Cox regression proportional hazards model for complication in the delayed two-stage reconstruction cohort before matching (N=1,347)

|  | Univariate | |  | |  | Multivariate | | |  | | |  |
| --- | --- | --- | --- | --- | --- | --- | --- | --- | --- | --- | --- | --- |
| Characteristics |  | **Hazard Ratio**  **(95% CI)** | | **P** | | | **Hazard Ratio**  **(95% CI)** | **P** | | |  |  |
| Year | 2018-2020  (vs. 2015-2017) | 1.05 (0.57-1.58) | | 0.651 | | |  |  | | |  |  |
| Age | > 45  (vs. ≤ 45) | 104 (0.76-1.42) | | 0.798 | | |  |  | | |  |  |
| Hypertension | Yes  (vs. No) | 2.11 (1.53-2.90) | | <0.001 | | | 2.14 (0.56-2.95) | <0.001 | | |  |  |
| Diabetes | Yes  (vs. No) | 1.84 (6.34-2.50) | | <0.001 | | | 1.97 (1.43-2.72) | <0.001 | | |  |  |
| Fractionation | HF  (vs. CF) | 1.20 (0.81-1.02 | | 0.237 | | |  |  | | |  |  |
| IMRT | Yes  (vs. No) | 0.91 (0.68-1.24) | | 0.580 | | |  |  | | |  |  |
| Bolus | Yes  (vs. No) | 0.72 (0.51-1.02) | | 0.068 | | | 0.75 (0.53-1.06) | 0.109 | | |  |  |
| Reconstruction material | Implant  (vs. Autologous) | 1.34 (0.46-2.56) | | 0.577 | | |  | | |  | | |
| Interval between PMRT and deﬁnitive surgery | 10 mon  (vs. ≤ 10 mon) | 1.00 (0.99-1.00) | | 0.628 | | |  | | |  | | |

Abbreviation: HF, Hypofractionated fractionation; CF, Conventional fractionation; IMRT, Intensity modulating radiotherapy

## **Supplementary Figure S1. Patient selection process**

Exclusions: n=53,359

- Multiple mastectomy (n=925)

- Radiotherapy initiated 1 year after mastectomy (n=7,950)

- Fraction <14 or > 35 (n=38,464)

- No reconstruction (n=6,020)

Exclusions: n=886

- Delayed 1 stage (n=400)

- Incomplete final reconstruction (n=366)

- Pre-PMRT complication (n=120)

## **Supplementary Figure S2. Complications incidence according to fractionation and reconstruction timing before matching**


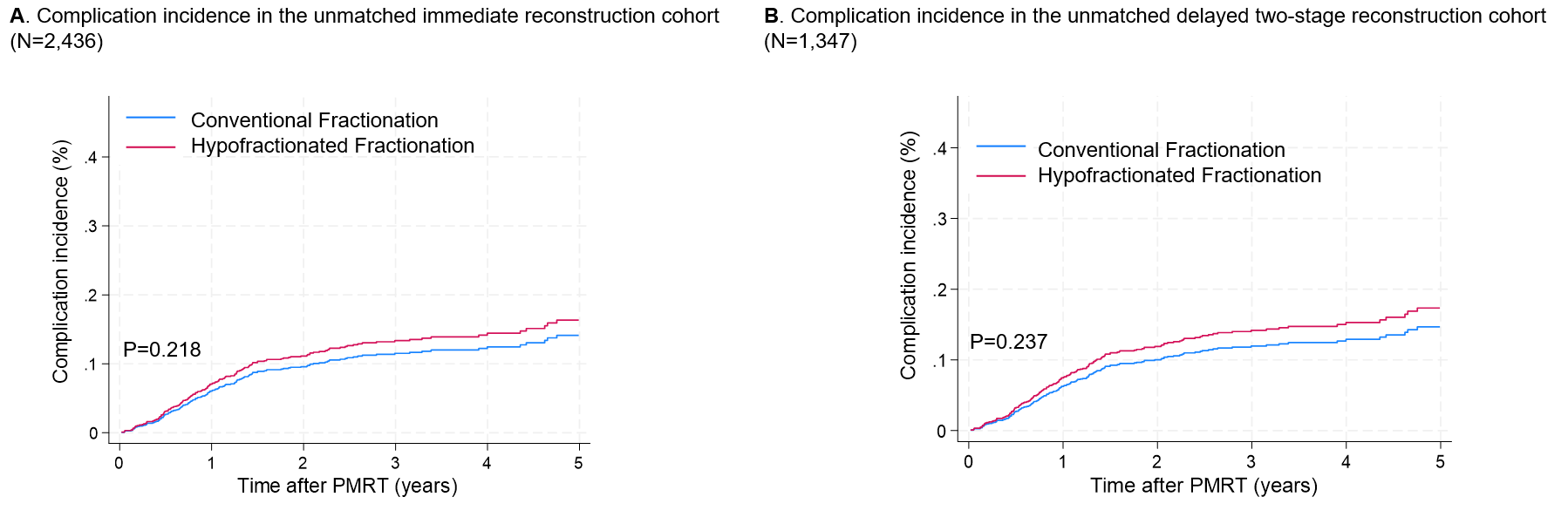


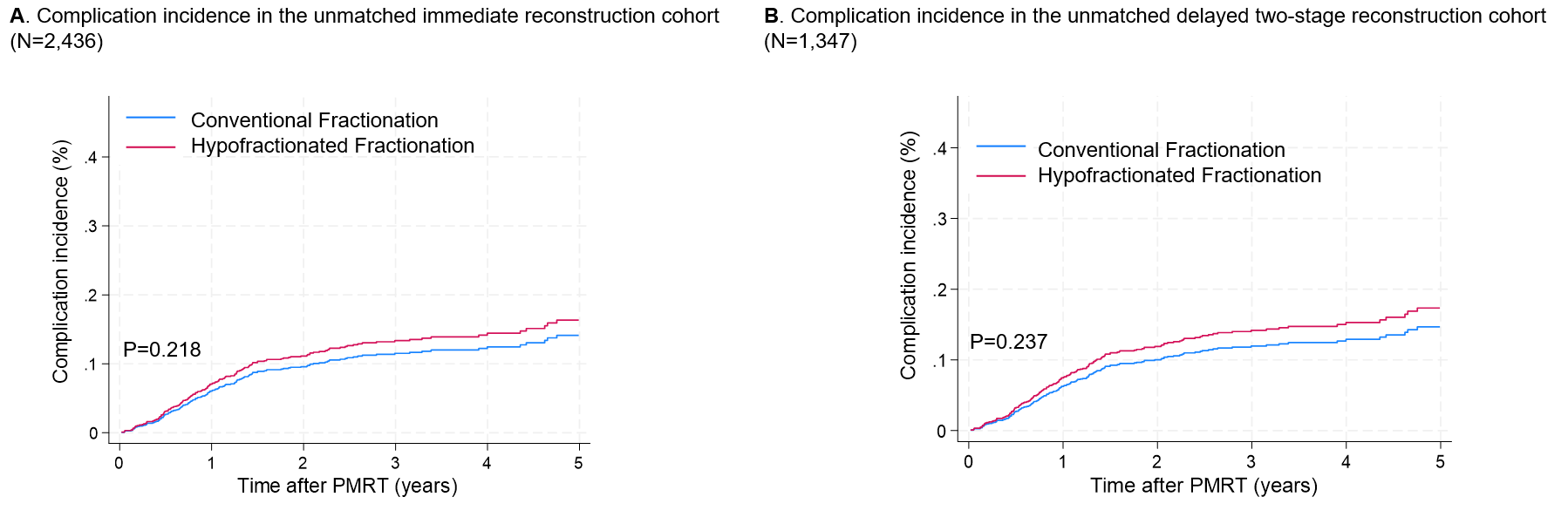

Supplement: Supplementary file 1 — Supplementray information [file 41416_2024_2741_MOESM1_ESM.docx]
